# The Perspective of Dysregulated LncRNAs in Alzheimer's Disease: A Systematic Scoping Review

**DOI:** 10.3389/fnagi.2021.709568

**Published:** 2021-09-21

**Authors:** Mohammad Reza Asadi, Mehdi Hassani, Shiva Kiani, Hani Sabaie, Marziyeh Sadat Moslehian, Mohammad Kazemi, Soudeh Ghafouri-Fard, Mohammad Taheri, Maryam Rezazadeh

**Affiliations:** ^1^Molecular Medicine Research Center, Tabriz University of Medical Sciences, Tabriz, Iran; ^2^Student Research Committee, Tabriz University of Medical Sciences, Tabriz, Iran; ^3^Student Research Committee, University of Social Welfare and Rehabilitation Sciences, Tehran, Iran; ^4^Department of Molecular Genetics, School of Biological Sciences, Tarbiat Modares University, Tehran, Iran; ^5^Department of Medical Genetics, Faculty of Medicine, Tabriz University of Medical Sciences, Tabriz, Iran; ^6^Department of Social Medicine, Zanjan University of Medical Sciences, Zanjan, Iran; ^7^Department of Medical Genetics, School of Medicine, Shahid Beheshti University of Medical Sciences, Tehran, Iran; ^8^Skull Base Research Center, Loghman Hakim Hospital, Shahid Beheshti University of Medical Sciences, Tehran, Iran

**Keywords:** Alzheimer's disease, lncRNAs, β-amyloid, NFT, BACE1-AS, NEAT1, MALAT1, SNHG1

## Abstract

LncRNAs act as part of non-coding RNAs at high levels of complex and stimulatory configurations in basic molecular mechanisms. Their extensive regulatory activity in the CNS continues on a small scale, from the functions of synapses to large-scale neurodevelopment and cognitive functions, aging, and can be seen in both health and disease situations. One of the vast consequences of the pathological role of dysregulated lncRNAs in the CNS due to their role in a network of regulatory pathways can be manifested in Alzheimer's as a neurodegenerative disease. The disease is characterized by two main hallmarks: amyloid plaques due to the accumulation of β-amyloid components and neurofibrillary tangles (NFT) resulting from the accumulation of phosphorylated tau. Numerous studies in humans, animal models, and various cell lines have revealed the role of lncRNAs in the pathogenesis of Alzheimer's disease. This scoping review was performed with a six-step strategy and based on the Prisma guideline by systematically searching the publications of seven databases. Out of 1,591 records, 69 articles were utterly aligned with the specified inclusion criteria and were summarized in the relevant table. Most of the studies were devoted to BACE1-AS, NEAT1, MALAT1, and SNHG1 lncRNAs, respectively, and about one-third of the studies investigated a unique lncRNA. About 56% of the studies reported up-regulation, and 7% reported down-regulation of lncRNAs expressions. Overall, this study was conducted to investigate the association between lncRNAs and Alzheimer's disease to make a reputable source for further studies and find more molecular therapeutic goals for this disease.

## Introduction

Alzheimer's disease (AD) is a disease known for its clinical symptoms, including gradual memory loss and language problems and cognitive impairments such as the inability to solve problems and spatial cognition and difficulty changing to mood (Cacace et al., 2016; Zhang and Wang, 2021). Accumulation of dense and insoluble beta-amyloid (Aβ) fragments outside and around neurons and neurofibrillary tangles (NFTs) resulted from the accumulation of hyper-phosphorylated Tau proteins inside cells are neuropathological symptoms of AD (Tiraboschi et al., 2004; McKhann et al., 2011). These lesions lead to neuronal degeneration, loss of synapses, and reduced neurotransmitter transport (Graham et al., 2017). AD dementia may affect 13.8 million Americans aged 65 and up by the middle of the century (Alzheimer's Association, 2020), and causes 50–75% of dementias (Association As, 2019). In terms of the time of onset, the disease is divided into two forms of early-onset AD (EOAD) and late-onset AD (LOAD). EOAD is diagnosed in patients under the age of 65, with a more significant genetic influence being reported for this form. LOAD accounts for 90% of cases seen in patients over 65 (Dursun et al., 2008; Wingo et al., 2012; Cacace et al., 2016). The genetic and etiological dimensions of the disease, focus on several specific genes, including amyloid precursor protein (APP), presenilin-1 (PSEN1), and presenilin-2 (PSEN2). Highly influential mutations in these genes can increase the susceptibility to AD, particularly the EOAD (Atri, 2019). Meanwhile, we should not forget the effect of non-coding RNAs in the pathogenesis of the disease.

Long non-coding RNAs (lncRNAs) are part of non-coding RNAs with sizes between 200 nucleotides and several kbs and high tissue specificity. They have fundamental role in regulation of gene expression (Zhou et al., 2021). According to the Annotations of the FANTOM5 project, about 28,000 lncRNA genes have been identified so far (Hon et al., 2017). Like mRNAs, lncRNAs are capped and polyadenylated and undergo the splicing process (Derrien et al., 2012). At the molecular level, lncRNAs play an essential role in transcription, translation, and regulation of gene expression, and chromatin remodeling and genomic imprinting (Statello et al., 2021), and at the biological level, they are one of the significant factors in the regulation of proliferation (Ponting et al., 2009), survival (Shen et al., 2015), and differentiation (Cesana et al., 2011). These non-coding RNAs are also involved in the pathogenesis and progression of AD due to their structural diversity and important biochemical properties (Idda et al., 2018).

Of the thousands of lncRNAs encoded in organs, around 40% of these lncRNAs are specifically expressed in brain tissue (Briggs et al., 2015). Many studies have shown an association between their expression dysregulation and many neurodegenerative diseases, including AD (Ni et al., 2017; Lyu et al., 2019). Studies performed on the 3xTg-AD model mice brain show that the expression of hundreds of lncRNAs is significantly changed compared with the control group (Zhou and Xu, 2015). Transcriptome analysis studies on human post-mortem brain tissues show changes in the expression levels of several lncRNAs in AD patients (Cao et al., 2019). Overall, both animal and human studies confirm the potential effect of lncRNAs on AD. To date, many studies have been done on AD and the physiopathology of the disease. On the other hand, more and more attention has been paid to lncRNAs, their structure, and their effect on AD development, progression, or treatment. In the present study, our focus has been on conducting a systematic scoping review of all clinical studies to summarize these studies and strengthen the link between the effect of lncRNAs on AD.

## Methods

### The General Framework for Review

The strategy for writing this article is based on the method proposed by Arksey and O'Malley (2005). This strategy was later improved by Levac et al. (2010) and Colquhoun et al. (2014). In this review, five steps of the 6-step framework are followed, which include:

1. Identifying the research question.
2. Search strategy.
3. Study selection.
4. Charting the data.
5. Collating, summarizing, and reporting the results.

Consultation is the optional sixth step and is not included in this article. The Preferred Reporting Items for Systematic Reviews and Meta-Analysis Extension for Scoping Reviews (PRISMA-ScR) Checklist is used to consider and observe two essential factors of clarity transparency in writing the article (Tricco et al., 2018).

### Identifying the Research Question

Our article was guided by the following questions in order to study, review, and discuss all original studies on lncRNAs in AD:

- What studies have been done on lncRNAs in AD?
- What are the results and findings of these studies?

### Search Strategy

Seven databases were searched for access to the publications: Pubmed, Scopus, Cochrane, Google Scholar, Embase, Web of Science, and ProQuest. The search did not apply a filter restricting the date, language, subject, or publication type. Review publications were also revised to reduce the possibility of missing related articles. “Alzheimer Disease” and “RNA, long non-coding” keywords were medical subject heading (MeSH) used in search strategy in PubMed and Embase database. The last search was conducted on APR 19, 2021. The references were managed using EndNote X8.1.

### Study Selection

Studies of AD concerning lncRNAs in humans, cell lines, and animal model studies were screened from publications obtained during the search process. All publication types were assessed, including journal articles, conference presentations, Erratum, conference abstracts, and reports. The screening was done in two stages by two reviewers (MRA, MH) separately. At this stage, the titles and abstracts of the articles were examined according to Table 1. The article's full text was reviewed, and irrelevant articles were deleted, and the articles remained utterly consistent with the research questions. Any contradiction in agreement with the opinion of the third person was resolved.

**Table 1:** Inclusion and exclusion criteria.

**Criterion**	**Inclusion**	**Exclusion**
Topic (disease, validating)	Alzheimer's disease	Non-Alzheimer's or unspecified dementia
	Using validating molecular techniques	Not-using validating molecular techniques
Study design	All study designs (original research)	Cross-sectional studies and commentaries
Language	English	Non-English
Time limit	All up to May 2021	—

### Charting the Data

After reaching the final articles that fulfill the research questions, we developed the data-charting. Study variables were created using the following headings: author's name, year of publication, country, type of study, human samples, animal models, cell lines, lncRNAs, methods, major findings, and references. Two reviewers (MRA, MH) separately extracted data from articles based on charts.

### Collating, Summarizing, and Reporting the Results

Quantitative and qualitative analysis was accomplished on the findings from the publications represented in tables and charts. A descriptive numerical summary of the extent, nature, and distribution of the studies was reviewed in the quantitative analysis section, and the presented data affirmation on the broader context suggested by Levac et al. (2010), conducted in a narrative review.

## Results

**Figure 1:**
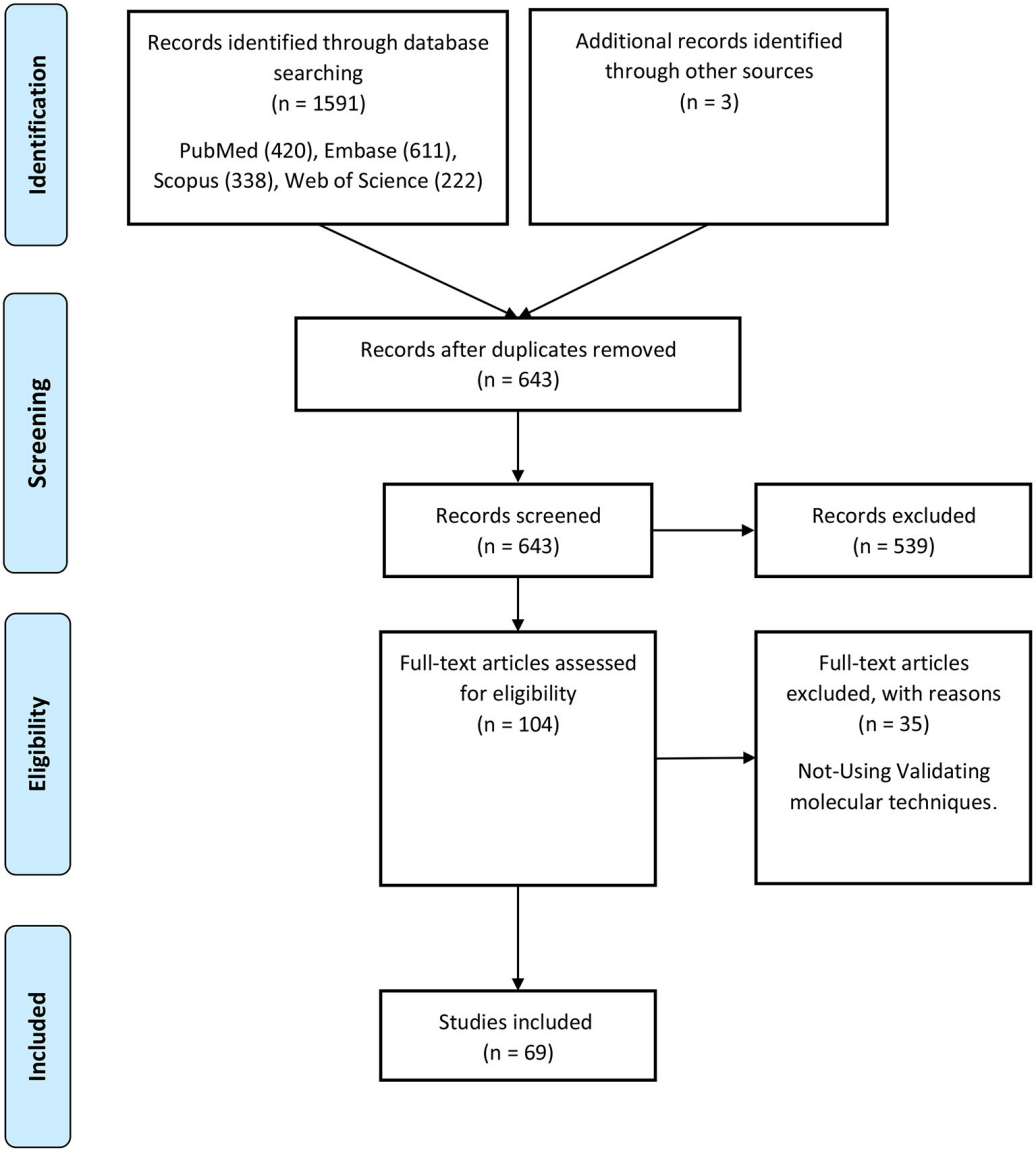
Flow chart of search strategy based on PRISMA flow diagram.

**Figure 2:**
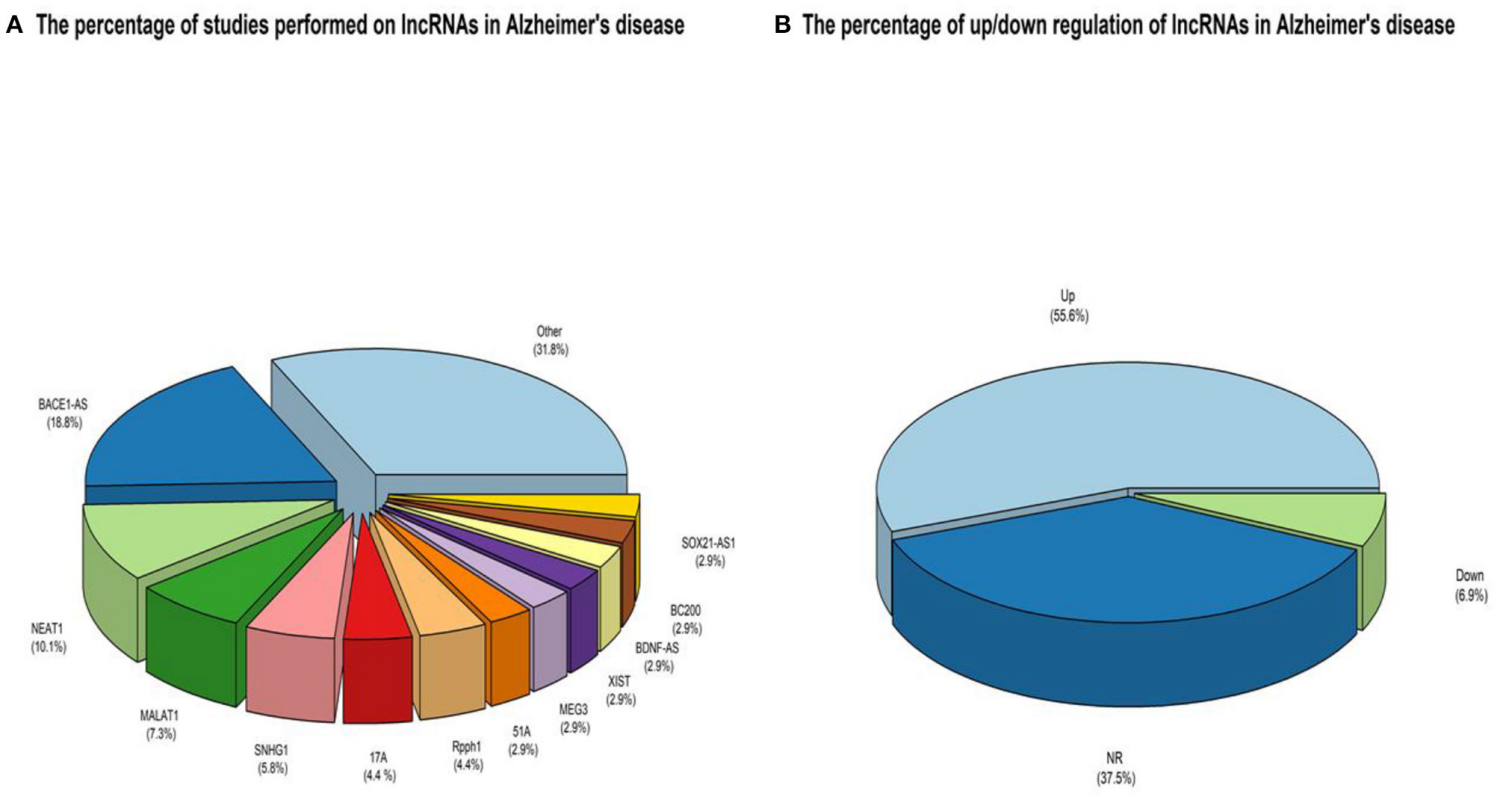
**(A)** An overview of the LncRNAs proportion is considered in the qualified studies. The other part is about lncRNAs that have only been studied once that can be accessed by referring to Table 3. **(B)** An overview of the ratio of up-regulated LncRNAs compared to down-regulated ones. NR also reflects non-reported studies of regulation levels.

A keyword search in seven databases yielded 1,591 records. In the meantime, three records were identified from other sources and added to the total number of articles. A total of 951 duplicate records were identified and deleted by Endnote software, and the total number of articles reached 643. After reviewing the titles and abstracts of the articles, 104 articles based on the research question were selected. Since it is impossible to select the exact desired studies from the abstract and the title alone, and the full text of the articles needs to be inspected, at this stage, by reviewing the full text of 104 articles, 69 articles were eligible to be included in Table 2 for the charting data stage. The process of selecting eligible articles and studies is described in detail in Figure 1. Eligible studies have been published from 2008 to 2021. Table 2 was designed to rank studies from top to bottom for faster access to article division based on the frequency of studies. Based on the mentioned number, 744 samples of AD patients and 771 healthy controls were included in these studies. In most cases, the sex of patients and controls is not mentioned. Mice were used as the model in 36 animal studies, and zebrafish was used in one study. There are 16 different cell lines used in these studies, including SH-SY5Y in 16 studies (Massone et al., 2011; Vaure and Liu, 2014; Huang et al., 2017, 2020; Cai et al., 2018; Li H. et al., 2018; Ke et al., 2019; Ma et al., 2019; Wang X. et al., 2019; Zeng et al., 2019; Zhang M. et al., 2019; Chen et al., 2020; Qasim et al., 2020; Wang Q. et al., 2020; Xu et al., 2020; Yan et al., 2020; Zhao et al., 2020; Zhou Y. et al., 2020; Zhang and Wang, 2021), HEK293 in 12 studies (Faghihi et al., 2008; Cai et al., 2017; Ghanbari et al., 2019; Ke et al., 2019; Zeng et al., 2019; Zhang et al., 2019; Zhu et al., 2019; Ge et al., 2020; Huang et al., 2020; Zhou B. et al., 2020; Zhang and Wang, 2021), PC12 in seven studies (Guo et al., 2018; Wang J. et al., 2018; Ma et al., 2019; Zhao et al., 2019; Bastard et al., 2020; Zhou B. et al., 2020; Zhang et al., 2021), SK-N-SH in 5 studies (Ke et al., 2019; Gao et al., 2020; Ge et al., 2020; He et al., 2020; Xu et al., 2020), N2A in five studies (Cai et al., 2017; Li D. et al., 2018; Butler et al., 2019; Huang et al., 2020; Yue et al., 2020), U251 in two studies (Lin et al., 2019; Zeng et al., 2019), Human peripheral neurons (HPNs) in two studies (Zeng et al., 2019; Ge et al., 2020), SK-N-F1 (Kang et al., 2014), RAW264.7 (Yamanaka et al., 2015), Hela (Spreafico et al., 2018), CHP212 (Gao et al., 2020), SK-N-AS (He et al., 2020), HT22 (Hong et al., 2020), BV2 (Zhang and Wang, 2021), and 20E2 (Ma et al., 2019) cell lines each were used in one study. The number and frequency of lncRNAs are shown in Figure 2. The following is a schematic view of the contribution of LncRNAs in studies and a comparison chart of up-regulated LncRNAs compared to down-regulated ones. Due to the large volume of methods and tests performed in these studies, only the major methods are mentioned. The distribution of studies is limited to only seven countries, in which China with 54 studies has a significant share (Vaure and Liu, 2014; Luo et al., 2015; Zhang et al., 2016, 2021; Cai et al., 2017, 2018; Deng et al., 2017; Fang et al., 2017; Huang et al., 2017, 2020; Yang et al., 2017; Feng et al., 2018; Guo et al., 2018; Li D. et al., 2018; Li H. et al., 2018; Liu et al., 2018; Wang J. et al., 2018; Wang X. et al., 2018, 2019; Zhang T. et al., 2018; Ke et al., 2019; Lin et al., 2019; Ma et al., 2019, 2020; Tang et al., 2019; Yi et al., 2019; Zeng et al., 2019; Zhang M. et al., 2019; Zhu et al., 2019; Bastard et al., 2020; Chen et al., 2020; Gao et al., 2020; Ge et al., 2020; He et al., 2020; Hong et al., 2020; Li et al., 2020; Qasim et al., 2020; Wang D. et al., 2020; Wang Q. et al., 2020; Xu et al., 2020; Yan et al., 2020; Yue et al., 2020; Zhao et al., 2020; Zhou B. et al., 2020; Zhou Y. et al., 2020; Zhuang et al., 2020; Zhang and Wang, 2021), followed by the United States with five studies (Faghihi et al., 2008; Airavaara et al., 2011; Kang et al., 2014; Yamanaka et al., 2015; Butler et al., 2019), Iran (Azizi-Aghaali et al., 2018; Fotuhi et al., 2019; Azadfar et al., 2020), and Italy (Massone et al., 2011; Spreafico et al., 2018; Garofalo et al., 2020) with three studies, and the Netherlands (Ghanbari et al., 2019) and Turkey (Kurt et al., 2020) and Israel (Banerjee et al., 2021) each with one study.

**Table 2:** Articles division.

**Type of studies**	**Percentage**	**References**
Cell culture, animal study	26.4%	Li D. et al., 2018; Wang X. et al., 2018; Butler et al., 2019; Lin et al., 2019; Ma et al., 2019; Zeng et al., 2019; Zhang et al., 2019, 2021; Bastard et al., 2020; Hong et al., 2020; Huang et al., 2020; Li et al., 2020; Qasim et al., 2020; Wang Q. et al., 2020; Yan et al., 2020; Yue et al., 2020; Zhao et al., 2020; Zhou B. et al., 2020
Cell culture	23.5%	Vaure and Liu, 2014; Cai et al., 2018; Li H. et al., 2018; Ke et al., 2019; Ma et al., 2019; Wang X. et al., 2019; Zeng et al., 2019; Zhang M. et al., 2019; Zhu et al., 2019; Chen et al., 2020; Gao et al., 2020; Ge et al., 2020; Gu et al., 2020; Xu et al., 2020; Zhou B. et al., 2020
Case-control	14.7%	Luo et al., 2015; Deng et al., 2017; Azizi-Aghaali et al., 2018; Feng et al., 2018; Guo et al., 2018; Fotuhi et al., 2019; Garofalo et al., 2020; Kurt et al., 2020; Wang D. et al., 2020; Zhuang et al., 2020
Animal study	13.2%	Zhang et al., 2016; Fang et al., 2017; Yang et al., 2017; Liu et al., 2018; Zhang T. et al., 2018; Yi et al., 2019; Azadfar et al., 2020; Ma et al., 2020; Banerjee et al., 2021
Case-control, cell culture, animal study	7.3%	Faghihi et al., 2008; Kang et al., 2014; Yamanaka et al., 2015; Ghanbari et al., 2019; Zhou Y. et al., 2020
Case-control, cell culture	7.3%	Massone et al., 2011; Spreafico et al., 2018; Wang J. et al., 2018; He et al., 2020; Zhang and Wang, 2021
Case-control, animal study	7.3%	Airavaara et al., 2011; Cai et al., 2017; Huang et al., 2017; Tang et al., 2019; Zhang M. et al., 2019

## The Perspective of Up-Regulated lncRNAs in AD

### BACE1-AS

Aβ plays an essential role in AD. The cleavage of APP causes the production of Aβ by β-secretase 1 (BACE1) and γ-secretase. Compared to normal individuals, BACE1 levels are increased in AD patients. Hence, increased BACE1 expression plays a critical role in AD (Zeng et al., 2019). Increasing the expression of some lncRNAs such as BACE1-AS induces BACE1 expression. BACE1-AS, as an antisense RNA, can positively regulate BACE1 mRNA and protein expression *in vivo* and *in vitro* (Faghihi et al., 2008; Zhang et al., 2018). BACE1-AS plays a crucial role in BACE1 stability through RNA duplex formation and can positively regulate BACE1 and protein expression (Zeng et al., 2019). The cortex of patients with AD showed significantly higher levels of HuD, and an increase in APP, BACE1, BACE1AS, and Aβ compared to the cortical tissue of healthy individuals (Kang et al., 2014).

Additionally, up-regulation of BACE1-AS leads to the prevention of the binding of miRNA to BACE1. The knockdown of BACE1-AS leads to an increase in the level of miRNAs, a reduction in the level of BACE1 expression (Zeng et al., 2019). Zhang et al. reported that BACE1-AS was significantly increased in the blood samples of patients with AD, and knockdown of BACE1-AS by siRNA increased the primary hippocampal neuron proliferation *in vitro*. BACE1-AS knockdown improved memory and learning behaviors in SAMP8 mice, inhibited BACE1, APP production, and tau protein phosphorylation in hippocampi (Zhang et al., 2018). In the Plasma of AD patients and SK-N-SH and SK-N-AS cells treated with Aβ and isoflurane, the BACE1-AS was upregulated, while miR-214-3p was downregulated. Additionally, miR-214-3p improved cognitive status in mouse models by preventing autophagy and reducing apoptosis *via* suppressing Atg12 expression (He et al., 2020). Therefore, BACE1-AS can play a critical role in the monitoring and management of AD.

### NEAT1

LncRNA nuclear enriched abundant transcript 1 (NEAT1) is highly evolutionarily conserved between humans and rodents, especially in the 5' region of the transcript (Hutchinson et al., 2007). Increased NEAT1 is associated with several cognitive and neurodegenerative disorders such as AD, schizophrenia, Huntington's, and Parkinson's. Studies in human and rodent samples have shown that NEAT1 may play an important role in neuroplasticity (Butler et al., 2019). NEAT1 is also involved in epigenetic regulation mechanisms in AD pathology (Lin et al., 2019). Wang et al. Found that NEAT1 interacts with the P300/CBP complex, and silencing of NEAT1 by suppressing acetyl-CoA production downregulated H3K27Ac and upregulated H3K27Cro level (Lin et al., 2019). Anderson et al. reported that NEAT1 is epigenetically involved in hippocampus-dependent, long-term memory formation, and knockdown of Neat1 resulted in extensive changes in gene expression and histone H3 lysine-9 dimethylations (H3K9me2) disturbances in the hippocampus of aged rodents (Butler et al., 2019). Huang et al. showed that NEAT1 is upregulated in the APP/PS1 transgenic mice and regulated the interaction between PINK1 and NEDD4L. Upregulation of NEAT1 induces ubiquitination and degradation of PINK1, leading to autophagy signaling, increased amyloid accumulation, and decreased cognition (Huang et al., 2020). In Aβ-treated SH-SY5Y and SK-N-SH cells, NEAT1 was increased, and its decrease inhibited Aβ-induced by reducing survival and p-Tau levels and promoting apoptosis. Also, NEAT1 acted as decoy and sponge of miR-107. miR-107 abundance was decreased in Aβ-treated cells (Ke et al., 2019). Hence, NEAT1 could provide new therapeutic approaches for AD.

### SNHG1

Small nucleolar RNA host gene 1 (SNHG1) is increased in various diseases and plays an oncogenic role in cancer (Gao et al., 2020). Silencing of SNHG1 promoted neuronal autophagy and prevented cell death in Parkinson's disease. Also, knockdown of SNHG1 has effectively prevented Aβ (25-35)-induced cell injury of SH-SY5Y and HPN cells (Wang H. et al., 2019). Gao et al. reported that SNGH1 could induce ZFN217 expression to modulate Aβ-induced cell injury by sponging miR-361-3p (Gao et al., 2020). Additionally, a study has shown that several lncRNAs and miRNAs, including SNHG1, are dysregulated in aged 2 × Tg-AD mice, and SNHG1 was targeted by Tet2 (Zhou B. et al., 2020).

### 17A

GABAB receptors (GABABR) are activated potassium channels and inhibit adenylate cyclase *via* G proteins. GABABR is a heterodimer of G protein-coupled receptors consisting of two subunits (GABABR1 and GABABR2). The GPR51 gene encodes GABABR2. LncRNA 17A strictly controls alternative splicing of GPR51. RNA polymerase III transcribes 17A from intron 3 of GPR51 (Massone et al., 2011; Luo and Chen, 2016). One study reported that a lack of 17A leads to inhibition of apoptosis, migration, and increased autophagy (Wang X. et al., 2019). Massone et al. showed that the expression of 17A was increased in the cerebral tissue of AD patients and demonstrated that its expression in neuroblastoma cells increased Aβ secretion in response to inflammatory stimuli. So, 17A may be a potential target for treating AD (Massone et al., 2011).

### 51A

SORL1 gene encodes SORLA protein, a receptor for apolipoprotein E, associated with AD (Motoi et al., 1999; Ciarlo et al., 2013). SORLA controls APP trafficking and processing and restricts Aβ peptide production. Allelic variants of the SORL1 gene are associated with AD disease, and the function of this gene is reduced in AD (Willnow and Andersen, 2013). 51A lncRNA is antisense of the SORL1 gene and is frequently increased in AD patients' cerebral cortices (Ciarlo et al., 2013). Ciarlo et al. reported that expression of 51A alters SORL1 splicing and shifts from the canonical long protein variant A to an alternatively spliced protein form. This process reduces the synthesis of variant A of SORL1, and with impaired APP processing, it leads to increased Aβ formation (Ciarlo et al., 2013). 51A expression has also been increased in the AD brain and *in vitro* models (Feng et al., 2018). However, the plasma level of lncRNA 51A did not show a significant difference between AD patients and healthy controls (Feng et al., 2018). This evidence suggests that 51A by reducing SORLA levels may be involved in AD progression, but more studies are needed in the future.

### XIST

The lncRNA X inactive specific transcript (XIST) is involved in developing many malignant tumors (Yue et al., 2020). XIST can act as an oncogenic lncRNA and induce growth in pancreatic and bladder tumors by interacting and inhibiting miR-124 (Liang et al., 2017; Xiong et al., 2017). It has been reported that miR-124 regulates the expression of BACE1 and is decreased in the AD tissues, implying that XIST might play a critical role in AD. Yue et al. Treatment of Na2 cells with H2O2 has created an AD model *in vitro*. Silencing of XIST has reduced the effect of H2O2 on miR-124, BACE1, and Aβ1–42 expression in N2a cells (Yue et al., 2020). A study in primary cultured rat hippocampal neurons showed that knockdown of XIST reduced Aβ25-35-induced neurotoxicity, apoptosis, and oxidative stress through upregulation of miR-132 (Wang X. et al., 2018). Therefore, XIST may be a new potential target therapy for AD (Yue et al., 2020).

### RPPH1

Ribonuclease P RNA component H1 (RPPH1) is part of the RNase P ribonucleoprotein RNA complex and converts precursor tRNA into mature tRNA by cleavage. RPPH1 enhances cdc45 expression levels and induces dendritic spine formation by binding to miR-330-5p (Cai et al., 2017). Gu et al. showed that the levels of rpph1 and miR-122 are increased in AD mice, and rpph1 by binding to miR-122 leads to the activation of the Wnt/β-catenin and Aβ-induced neuronal apoptosis in SH-SY5Y cells (Qasim et al., 2020). Also, Aβ25-35-induced apoptosis and ER stress in SH-SY5Y cells could be reduced by RPPH1. RPPH1 targets miR-326; thereby, the inhibitory effect of miR-326 on Pyruvate kinase M2 (PKM2) is removed. PKM2 regulates cell death and apoptosis by modulating glycolysis metabolism. Therefore, RPPH1 could be involved in AD (Gu et al., 2020).

### TUG1

Taurine Upregulated Gene 1 (TUG1) encodes a new lncRNA that is 6.7 kb in length and is located on chromosome 22q12. At first, the essential role in retinal development and the formation of photoreceptors was identified (Lin et al., 2016). It was later found that TUG1 promotes apoptosis by sponging miR-9 and up-regulation of BCL2L11 under ischemia. Up-regulation of TUG1 is associated with the pathogenesis of Huntington's disease, which is a neurodegenerative disease (Chen et al., 2017). Li et al. Reported that knockdown of TUG1 inhibits the apoptosis of hippocampal neurons in AD by upregulating miR-15a and downregulating ROCK1 expression. Therefore, it may serve as a new therapeutic target in AD (Li et al., 2020).

### LoNA

Long nucleolus-specific lncRNA (LoNA) reduces rRNA production by reducing nucleolin (NCL) transcription and decreases rRNA 2′-O-methylation by reducing active fibrillarin (FBL). The 5' portion of LoNA has NCL binding site, and the 3' portion of LoNA has a snoRNA for binding to FBL (Decatur and Fournier, 2002; Li D. et al., 2018). Decreased LoNA leads to increased rRNA and ribosome levels and increased translation. Also, the transport of ribosomes to synapses is enhanced, leading to increasing AMPA/NMDA receptors, synapse flexibility, and ultimately enhancing long-term memory. Knockdown of LoNA, in addition to increasing long-term memory in WT mice, improved memory function in APP/PS1 transgenic mice (Li D. et al., 2018).

### SOX21-AS1

The Wnt signaling pathway is involved in the proliferation, differentiation, and survival of neuronal cells (Kishimoto et al., 2008). Traces of this pathway have also been identified in carcinogenesis and neurodegenerative disorders such as AD (Inestrosa et al., 2007). SOX21-AS1 is increased in AD patients. Silencing of SOX21-AS1 in AD mice could reduce neuronal oxidative stress and inhibit apoptosis in neuronal cells by upregulation of FZD3/5 and activating the Wnt pathway. Frizzled 3/5 (FZD3/5) are two receptors required for the Wnt signaling pathway, which play a role in developing the central nervous system, including synaptogenesis and structural plasticity (Zhang et al., 2019). Therefore, future studies can assess SOX21-AS1 as a new target for AD treatment.

### BC-200

LncRNA BC-200 encodes from Brain Cytoplasmic RNA 1 (BCYRN1) gene by RNA pol III. The BC-200 is a translational regulator that targets the eukaryotic initiation factor 4A (eIF4A), maintaining long-term synaptic plasticity. BC-200 levels in the brains of AD patients are increased (Li H. et al., 2018). However, in 2007, a study reported a decrease in its expression (Mus et al., 2007). This discrepancy may be due to differences in brain regions and the severity of the disease. In a study of post-mortem specimens in the control group, BC-200 levels were reduced. However, in AD brains, compared with normal brains, BC-200 levels were significantly up-regulated (Ahmadi et al., 2020). Li et al. showed that the expression BC-200 and BACE1 are increased in Aβ1-42 induced AD cell model. They also reported that inhibition of BC-200 by targeting and suppressing BCAE1 expression reduced apoptosis and increased cell viability in AD cells. So BC-200 could provide new insights into AD gene therapy (Li H. et al., 2018).

### BDNF-AS

BDNF is involved in neurogenesis and synaptic plasticity, and its decrease in the brain led to damage to memory and learning. Levels of BDNF are decreased in patients with advanced and mild AD (Azizi-Aghaali et al., 2018). LncRNA BDNF-AS is an antisense transcript to BDNF and could negatively regulate BDNF (Guo et al., 2018). Real-time PCR data showed a significant increase in BDNF-AS levels in the plasma of patients compared to controls (Azizi-Aghaali et al., 2018). Guo et al. reported that in Aβ25-35-induced PC12 cells, BDNF-AS is increased, but BDNF is decreased. These expression changes promote apoptosis and reduce cell viability. Additionally, silencing of BDNF-AS increases the cell viability and inhibits oxidative stress and apoptosis of Aβ25-35-induced PC12 cells through upregulation of BDNF (Guo et al., 2018).

### ANRIL

Lnc-antisense non-coding RNA in the INK4 locus (lnc-ANRIL) is located on chromosome 9 and regulates neuronal functions and inflammation. A study in diabetic rats revealed that silencing of this lncRNA inhibited the NF-κB signaling pathway and subsequently improved memory and reduced apoptosis of hippocampal neurons (Wen et al., 2018). Inflammation is involved in the pathogenesis of AD, and lnc-ANRIL can regulate inflammation and cytokine expression through association with the NF-κB or other inflammatory pathways such as the BRCC3 signaling pathway. Zhou et al. reported that ANRIL silencing increases neurite outgrowth, suppresses cell apoptosis and inflammation by binding to miR-125a in the Pc12 cell line. Therefore, ANRIL may be a potential therapeutic target for AD (Zhou B. et al., 2020).

### LncRNA-ATB

Dysregulation of the lncRNA activated by transforming growth factor-β (lncRNA ATB) involves various pathological processes, such as colorectal cancer and pancreatic cancer (Yue et al., 2016). There are limited reports on the role of lncRNA ATB in neurodegenerative diseases such as AD. The role of lncRNA ATB in Aβ25-35-induced PC12 cell injury has been investigated. The results showed that in AD patients, lncRNA ATB expression is increased. In P12 cells, lncRNA ATB negatively regulates the expression of miR-200, and miR-200 can negatively regulate ZNF217. Thus, suppression of lncRNA ATB reduced Aβ25-35-induced PC12 cell injury by regulating the miR-200/ZNF217 axis (Wang J. et al., 2018).

## The Perspective of Down-Regulated lncRNAs in AD

### MALAT1

Metastasis-associated lung adenocarcinoma transcript 1 (MALAT1) is abundantly expressed in neurons. MALAT1 is involved in synaptic density (Wu et al., 2013), Schwann cell proliferation and migration, and in initiating regenerative responses after peripheral nerve injury. Also, MALAT1 has the potential to protect neurons and modify anti-inflammatory effects, and it possibly plays a protective role in AD pathology. Ma et al. reported that MALAT1 boosts neurite outgrowth and prevents neuron apoptosis and inflammation in AD through interaction with miR-125b (Ma et al., 2019). Also, MALAT1 can act as a sponge for miR-30b and increase CNR1 expression, which stimulates PI3K and AKT phosphorylation and ultimately could improve neuronal recovery following AD in animal and cell models (Bastard et al., 2020). These studies suggested the critical role of MALAT1 in neuronal loss and inflammation.

### LncRNA MEG3

The PI3K/Akt pathway plays an essential role in protecting neurons and inhibiting apoptosis by increasing SOD expression. This pathway appears to be vital in AD because it is related to hyper-phosphorylated tau protein (Matsuda et al., 2018). Maternally expressed gene 3 (MEG3) lncRNA is involved in PI3K/Akt pathway. Yi et al. reported that MEG3 expression is decreased in the tissues of AD rats. Also, upregulation of MEG3 led to improved cognitive impairment, reduced neuronal damage, reduced Aβ positive expression, and inhibits activation of astrocytes in hippocampus tissues in AD rats *via* inactivation of the PI3K/Akt signaling pathway (Yi et al., 2019). Therefore, increased expression of MEG3 may lead to an improvement in AD.

### WT1-AS

Another notable lncRNA associated with AD is WT1-AS. Wang et al. reported low expression of WT1-AS in cell models induced by Aβ25-35. Overexpression of WT1-AS through inhibition of WT1 expression can suppress miR-375 expression and promote SIX4 expression, thus preventing neuronal apoptosis and oxidative stress injury (Wang Q. et al., 2020).

### Other lncRNAs

LRP1-AS is another lncRNA that is dysregulated in AD. LRP1 locus produces both LRP1 mRNA and a spliced LRP1-AS of the LRP1 gene. LRP1 plays a role in the systemic clearance of AD amyloid-beta (Aβ), and LRP1 expression levels are critical for AD progression. Yamanaka et al. reported that in the AD brain, Lrp1-AS expression is increased and negatively regulates the expression of Lrp1. Lrp1-AS directly binds to Hmgb2 and inhibits Hmgb2 activity to increase Srebp1a-dependent Lrp1 transcription (Yamanaka et al., 2015). In a recent study, MAGI2-AS3 is significantly increased in AD patients and act as a sponge and negative regulator for miR-374b-5p (Zhang and Wang, 2021). Also, they reported that decreased MAGI2-AS3 expression and increased miR-374b-5p expression reduce Aβ-induced neurotoxicity and inflammation. The results of luciferase activity provide evidence for the interaction of miR-374b-5p with BACE1 (Zhang and Wang, 2021). Therefore, the MAGI2-AS3/miR-374b-5p axis can be considered as a biomarker for AD (Zhang and Wang, 2021). Linc00507 was significantly increased in AD mice and AD-like SH-SY5Y cells. linc00507 through binding to miR-181c-5p regulates expression of microtubule-associated Tau protein (MAPT) and microtubule tau tubulin kinase (TTBK1). Also, linc00507 can mediate tau protein hyperphosphorylation by activating the P25/P35/GSK3β signaling pathway through MAPT/TTBK1 regulation (Yan et al., 2020). It has recently been reported that RP11-543N12.1 enhanced apoptosis and suppresses an AD cell model's proliferation *via* targeting miR324-3p. Thus, it is suggested that RP11-543N12.1 and miR-324-3p may serve as practical biomarkers and therapeutic targets for AD in the future (Cai et al., 2018). Additional studies have been conducted on the role of other lncRNAs in AD, such as LINC00094, RP11-543C4.3-001, GDNFOS, n336694 (Airavaara et al., 2011; Huang et al., 2017; Zhu et al., 2019; Chen et al., 2020) (Table 3).

**Table 3:** LncRNAs in Alzheimer's disease.

**References**	**Country**	**Type of study**	**Human sample(s)**	**Animal model(s)**	**Cell line(s)**	**lncRNA(s)**	**Up or down**	**Major method(s)**	**Major findings**
Faghihi et al. (2008)	USA	Cell culture case-control animal study	AD patients (40 cases and 40 controls)	Tg19959 male mice	HEK293T	BACE1-AS	up	Enzyme complementation assay. Human samples, RT-PCR, Mouse studies	BACE1-AS expression is increased in AD patients and disease model mice. BACE1-AS expression is increased by various stressors such as Aβ 1–42, increasing BACE1 mRNA stability and further Aβ 1–42.
Airavaara et al. (2011)	US	Case-control Animal study	Post mortem MTG samples of controls, AD, and HD	Sprague-Dawley rats	-	GDNFOS		Quantitative RT-PCR Western Blot	Dysregulation of GDNF and DNSP-11 and GDNFOS may have played a role in AD pathogenesis
Massone et al. (2011)	Italy	Case-control Cell culture	NA	-	SHSY5Y	17A	up	*In vitro* transcription, Primer extension reaction, Q-RT PCR, Immunoprecipitation, Western blot, Measurement of cAMP accumulation, Patch-clamp, Aβ detection in SHSY5Y permanently transfected cells	17A lncRNA is up-regulated in the brain tissue of AD patients and increases the secretion of beta-amyloid peptides. Synthesis of 17A can be induced by inflammation.
Kang et al. (2014)	USA	Cell culture Case-control Animal study	AD patients (20 cases and 20 controls)	HuD-Tg mice	SK-N-F1	BACE1-AS	up	Cell Culture, siRNA, and Plasmids, Protein Analysis, RNA Analysis	The level of HuD expression in AD patients' brains is higher than in the controls and the brains of HuD. Tg mice have higher expression levels of APP, BACE1, and BACE1-AS. HuD increases APP production and increases cleavage to Aβ fragments.
Vaure and Liu (2014)	China	Cell culture	-	-	SH-SY5Y	BACE1-AS	up	Aβ1-42 treatment, MTT assay, qPCR, Western blot, IF staining, ELISA assay, Ribonuclease protection assay, siRNA, and cell transfection	Down-regulation of BACE1 - AS by siRNA decreased BACE1's ability to cleavage APP and delayed SP plaques' formation.
Luo et al. (2015)	China	Case-control	AD and MCI patients [106 cases (AD)and 67 cases (MCI)] and 179 controls)	-	-	linc01080		Study population, DNA extraction, SNP genotyping	No difference was found between allele frequency in the SNP rs7990916 between patients and controls.
Yamanaka et al. (2015)	USA	Case-control Cell culture Animal model	NA	Mice	RAW264.7	LRP1-AS	up	Cell Culture, Animal Studies, qRT-PCR, RNase-Assisted RNA Chromatography, WB and IP, Luciferase Reporter Assays, ChIP	In the Alzheimer's brain, Lrp1-AS expression increases, and Lrp1 expression decreases. Lrp1-AS binds directly to Hmgb2 and inhibits Hmgb2 activity to increase Srebp1a-dependent Lrp1 transcription.
Zhang et al. (2016)	China	Animal model	-	Mice	-	ENSMUST00000187351.1 ENSMUST00000193125.1 ENSMUST00000198676.1 TCONS_01857304 TCONS_03323270 TCONS_00506853 TCONS_03830561 TCONS_02311112		RNA sequencing, qPCR, Functional enrichment analysis: GO and KEGG	This study provided a catalog of SAMP8 brain lncRNA mice further to understand their regulatory role in AD's pathogenesis. lncRNAs, along with their application in other diseases, have become effective therapeutic targets.
Cai et al. (2017)	China	Cell culture Animal study	-	B6C3-Tg (APPswe, PSEN1dE9) 85Dbo/Mmjax mice C57BL/6J mice (control)	Neuro-2a cells HEK 293T	Rpph1	up	qRT-PCR, whole transcriptome seq, western blot	Rpph1 binds to miR326-3p/miR-330-5p and leads to CDC42 upregulation. Upregulation of Rpph1 increased dendritic spine density in primary cultured hippocampal pyramidal neurons, whereas downregulation of Rpph1 had the reverse effect.
Deng et al. (2017)	China	Case-control	AD patients (70 cases and 90 controls)	-	-	51A	up	qRT-PCR	lncRNA 51A is up-regulated in patients with AD compared to controls and is stable in Plasma.
Fang et al. (2017)	China	Animal study	-	APP/PS1 mice	-	Gm13498 DQ113493 AK038159 1700 030L20Rik		Microarray, qRT-PCR	LncRNA 1700030L20Rik and lncRNA Gm13498 may block its translocation into the nucleus by binding to Rest protein, leading to reduced Rest expression and the loss of neuroprotective effect of Rest.
Huang et al. (2017)	China	Cell culture Animal study	-	PPswe/PS1E9 (APP/PS1) mice	SH-SY5Y	lnc RNA n336694	up	Real-time PCR, Western blotting	lnc RNA n336694 and miR-106b were overexpressed in APP/PS1 mice brain tissues.
Yang et al. (2017)	China	Animal study	-	Sprague-Dawley rats	-	315 lncRNAs Such as: BC158567, MRAK050857, MRuc009dux S69385, XR_008107, MRAK081790	Down (BC158567, MRAK050857, MRuc009dux) Up (S69385, XR_008107, MRAK081790)	Microarray Analysis, qRT-PCR	Three hundred fifteen lncRNAs and 311 mRNAs were significantly dysregulated in the AD model.
Azizi-Aghaali et al. (2018)	Iran	Case-control	AD patients (30 cases and 30 controls)	-	-	BDNF-AS	Up	qRT-PCR	lncRNA BDNF-AS is present in the Plasma of patients and controls but is up-regulated in patients with AD.
Cai et al. (2018)	China	Cell culture	-	-	SH-SY5Y	RP11-543N12.1	up	Chip hybridization, RT-qPCR, Western blot, Dual-luciferase reporter assay, ELISA, MTT assay	RP11-543N12.1 enhanced the apoptosis and suppressed the proliferation of an AD cell model *via* targeting of miR 324-3p.
Feng et al. (2018)	China	Case-control	AD patients (80 cases and 72 controls)	-	-	17A 51A BACE1-AS BC200	Up (BACE1-AS)	qRT-PCR	The plasma level of four LncRNAs was compared between AD and non-AD patients and determine that BACE1 levels were increased in the plasma of AD patients and have high specificity for AD.
Guo et al. (2018)	China	Cell culture	-	-	PC12	BDNF-AS	up	qRT-PCR, Western blot	Silencing of BDNF-AS increases the cell viability, inhibit oxidative stress and apoptosis of Aβ25-35-induced PC12 cells through regulation of BDNF.
Spreafico et al. (2018)	Italy	Case-control Cell culture	AD patients (10 cases and 11 controls)	-	HeLa Cells	NEAT1 HOTAIR MALAT1		Cell Cultures, Antisense Oligonucleotides Transfection, qRT-PCR	In oligonucleotide transfection, the expression levels of NEAT1, HOTAIR, and MALAT1 decreased by 61, 71, and 78%, respectively. Because CDK5R1 expression is negatively regulated by NEAT1 and HOTAIR, turning them off increased CDK5R1 expression. CDK5R1 expression level increased with MALAT1 silencing.
Li H. et al. (2018)	China	Cell culture	-	-	SH-SY5Y	BC200	Up	qRT-PCR, Western blot, MTT assay, Flow cytometer	Inhibition of BC200 by targeting and suppressing BCAE1 expression reduced apoptosis and increased cell viability in AD cells.
Wang J. et al. (2018)	China	Case-control Cell culture	AD patients (18 cases and 16 controls)	-	PC12	lncRNA-ATB	up	MTT assay, Flow cytometry, LDH assay, Luciferase reporter assay, qRT-PCR, Western blot	In AD patients, lncRNA-ATB expression is increased. Suppression of lncRNA-ATB by regulating the miR-200 / ZNF217 axis protects PC12 cells against Aβ25-35-induced neurotoxicity.
Wang X. et al. (2018)	China	Cell culture Animal model	-	Sprague-Dawley rat embryos	Rat embryo Primary hippocampal neurons	XIST	up	Cell culture Aβ25-35 treatment, qRT-PCR, Cell transfection, MTT assay, LDH release assay, TUNEL, Western blot, Luciferase reporter assay	XIST expression is increased in hippocampal neurons as a result of the Aβ25-35 treatment. Knockdown of XIST improves toxicity, oxidative stress, and apoptosis induced by Aβ25-35 treatment in hippocampal neurons by targeting miR-132.
Zhang T. et al. (2018)	China	Animal study	-	Tg2576-APPswe mice	-	BC1		IHC, qPCR, RNA FISH Assay, EMS Assay	Inhibition of BC1 or BC1-FMRP in AD mice blocks Aβ aggregation in the brain and protects against memory deficits and spatial learning. Expression of exogenous BC1 in mice's excitatory pyramidal neurons induces Aβ peptides accumulation and memory impairments, and spatial learning.
Li D. et al. (2018)	China	Cell culture Animal model	-	C57BL/6J mice	N2a	LoNA		FISH and immunostaining, Northern blots and densitometry analysis, RNA pull-down, nuclear run-on (NRO) analysis	The vital role of LoNA in modulating ribosome production in response to translation demands in long-term memory was confirmed.
Liu et al. (2018)	China	Animal study	-	SAMR1 and SAMR8 mice	-	BACE1-AS	up	RT-qPCR, Western blot, ELISA	BACE1-AS was significantly increased in the blood samples of patients with AD, and knockdown of BACE1-AS by siRNA increased the primary hippocampal neuron proliferation *in vitro*. Also, BACE1-AS knockdown mediated by lentivirus improved memory and learning behaviors in SAMP8 mice, inhibited BACE1, APP production, and phosphorylation of tau protein.
Wen et al. (2018)	China	Animal model AND Cell line	-	Mouse (APPswe /PS1dE9)	SH-SY5Y	EBF3-AS		Gene knockdown (siRNA) Real-time PCR Western blot	LncRNA EBF3-AS induced neuron apoptosis in AD and play a role in EBF3 expression regulation.
Butler et al. (2019)	United States	Cell culture Animal study	-	C57BL/6 mice	N2a cell	NEAT1	up	CRISPR, Western blotting, Reverse transcription qPCR, ChIP, RIP	Overexpression of NEAT1 using CRISPRa resulted in memory impairment in young adult mice, while decrease NEAT1 in young and old adult mice improved memory. These results suggest that lncRNA NEAT1 is a hippocampal-dependent epigenetic suppressor and plays a vital role in long-term memory formation.
Fotuhi et al. (2019)	Iran	Case-control	AD patients (45 cases and 36 controls)	-	-	BACE1-AS	Down	Exosomes Purification, SEM Analysis, Size Distribution Analysis, Purification of Total RNA from the Plasma and the Plasma-Derived Exosomes Samples, qRT-PCR, ApoE Genotyping	BACE1-AS expression levels decreased in the pre-AD subgroup's Plasma and increased in AD patients' Plasma relative to controls. Roc curve analysis can differentiate between pre-AD patients and healthy controls with a sensitivity of 75%, between full-AD patients and healthy controls with a sensitivity of 68%, and between pre-AD and full-AD patients with a sensitivity of 78%.
Ghanbari et al. (2019)	Netherlands	Cell culture Case-control Animal study	AD patients	miR-142-/- knockout mouse	HEK293 neural progenitor cells (NPC)	BZRAP1-AS1		GWAS study on AD, q-PCR, Putative target genes of miR-142, RNA-Seq analysis in NPC, RNA-Seq analysis in the hippocampus of miR-142 KO mice and wt littermates,	The rs2526377: A> G variant of BZRAP1 - AS lncRNA is associated with a reduced risk of AD.
Ke et al. (2019)	China	Cell culture	-	-	SH-SY5Y SK-N-SH HEK293T	NEAT1	Up	RT-qPCR MTT assay, RIP assay, Western blot, Flow cytometry, luciferase activity	NEAT1 expression was upregulated in Aβ-treated SH-SY5Y and SK-N-SH cells. Knockdown of NEAT1 reduced Aβ-induced neuronal injury by sponging miR 107.
Ma et al. (2019)	China	Cell culture	-	-	PC12	MALAT1		MTT assay, RT-qPCR, Western blot, luciferase Reporter	Inc-MALAT1 interacts with miR-125b, prevent inflammation and neuron apoptosis While inducing neurite outgrowth in AD.
Wang H. et al. (2019)	China	Cell culture	-	-	SH-SY5Y HEK293 HPNs	SNHG1	up	cell culture, Aβ 25-35 preparation, qRT-PCR, Western blot assay, MTT assay, Flow cytometry, MMP, Caspase-3 activity assay, Luciferase reporter assay	The results showed the up-regulation of SNHG1 in the *in-vitro* cell model of AD. SNHG1 knockdown was impactful in preventing Aβ25-35-induced cell injury of SH-SY5Y and HPN cells.
Wang X. et al. (2019)	China	Cell culture	-	-	SH-SY5Y	17A	Up	q-RT PCR, Western blot, Flow Cytometry, Immunofluorescence, ELISA assay	17A-overexpressing induces autophagy and neurodegeneration and also deactivates GABAB signaling.
Lin et al. (2019)	China	Cell culture Animal study	-	(APPswe/PS1dE9)	U251	NEAT1		RNA seq, Western blot, Luciferase assay, Flow cytometry	NEAT1 is involved in epigenetic regulation mechanisms in AD pathology. NEAT1 interacts with the P300/CBP complex and silencing of NEAT1 by suppressing acetyl-CoA production downregulated H3K27Ac and upregulated H3K27Cro level.
Yi et al. (2019)	China	Animal study	-	Sprague-Dawley rats	-	MEG3	Down	IHC, Western blot, TUNEL, RT-qPCR	Increasing the lncRNA MEG3 reduces neuronal damage and cognitive impairment. Also, upregulation of the lncRNA MEG3 inhibits the activation of astrocytes in hippocampal tissues in AD by inhibiting the PI3K/Akt signaling pathway.
Tang et al. (2019)	China	Case-control Animal study	AD patients (3 cases and 3 controls)	C57BL/6J mice	-	AK045227 AK013093 AK080003 AK037309		Morris Water Maze Test, ELISA (The level of pro-inflammatory cytokines), IHC, Western Blot, Microarray Analysis, qRT-PCR	Specific lncRNAs between patients and controls played a significant role in inflammation, apoptosis. FOXL1, CDC5L, ONECUT2, and CDX1 are among the major transcription factors regulating the expression of these lncRNAs.
Zeng et al. (2019)	China	Cell culture Animal study	-	APP/PS1 transgenic mice	HEK293T SH-SY5Y U251	BACE1-AS	Up	RIP assay, Western blot, Real-time PCR, Dual-luciferase assay	Overexpression of BACE1-AS prevents the degradation of BACE1 mRNAs by sponging the miRNAs that target BACE1.
Wang X. et al. (2019)	China	Cell culture Animal model	-	specific pathogen-free (SPF) Kunming (KM) mice	HEK-293 T	SOX21-AS1		Microarray, IHC, Dual-Luciferase Reporter Gene Assay, RT-qPCR, Western Blot, Flow Cytometry	Silencing of the SOX21-AS1 lncRNA could reduce neuronal oxidative stress and suppress neuronal apoptosis in AD mice.
Zeng et al. (2019)	China	Cell culture	-	-	SH-SY5Y cells	Approximately 100 lncRNA (SNHG1, RN7SL1, SCARNA9, SNHG16, RGS5, AGAP2-AS, LINC01963)		RNA-seq Differential lncRNA expression analysis RT-qPCR	Approximately 100 dysregulated lncRNA were found in Aβ-treated SH-SY5Y cells, for instance, upregulation of SNHG1, RN7SL1, SCARNA9 and downregulation of SNHG16, RGS5, AGAP2-AS, LINC01963. Therefore, these lncRNAs may play a critical role in AD pathology through altered signal pathways.
Zhang M. et al. (2019)	China	Cell culture Animal study	-	C57/BL6J mice	PC-12	NEAT1	Up	qRT-PCR, FACS (flow cytometry), Luciferase assay, Western blot	NEAT1 promotes the development of AD by regulating the miR-124/BACE1 axis.
Zhu et al. (2019)	China	Cell culture	-	-	hCMEC/ D3 HBVP NHA HEK293T	LINC00094	up	RT-qPCR, microarray, Western blot, Horseradish peroxidase (HRP) flux, Immunofluorescence assays, TEER assays, Luciferase reporter assay, RIP assay	In Aβ1-42-incubated ECs, the expressions of LINC00094 and Endophilin-1 were increased, and the expressions of miR-224-5p/miR-497-5p were decreased. Also, the Silencing of LINC00094 promotes MEM's effect on decreasing blood-brain barrier permeability in the AD microenvironment.
Azadfar et al. (2020)	Iran	Animal study	-	Wistar rats (ICV-STZ rats)	-	BACE1-AS		RT-qPCR, ELISA	The level of the Bace1 protein can be helpful as a biomarker for prognosis, and Bace1-as expression can be used during the AD progression.
Chen et al. (2020)	China	Cell culture	-	-	SH-SY5Y cells	RP11-543C4.3-001		Detection of the Expression of Long Intronic Non-coding RNA and *CYP46A1*, Measurement of Ab and 24-OHC Content, Dual-Luciferase Assays	LincRNA overexpression inhibits cyp46a1 gene expression and inhibits the production of 24-OHC and beta-amyloid. Genotype A has a more robust gene inhibitory function than genotype G of the rs754203 variant located in the Linc sequence.
Gao et al. (2020)	China	Cell culture	-	-	SK-N-SH CHP212	SNHG1	Up	Cell counting kit 8 (CCK8) assay, qRT-PCR, Flow cytometry, Western blot analysis, ELISA, Dual-luciferase reporter assay, RIP assay	SNHG1 increases cell damages during the regulatory axis miR361-3p/ZNF217. Knocking down SNHG1 reduces the pathological effects of Aβ.
Garofalo et al. (2020)	Italy	Case-control	AD patients (six cases and six controls)	-		CH507-513H4.4 CH507-513H4.6 CH507-513H4.3	up	Isolation of Human Peripheral Blood Mononuclear Cells and RNA Extraction Sequencing, Pathway Analysis, RT-qPCR	Twenty-three genes were identified as differentially expressed, of which 3 LNCs were reported as up-regulated.
Ge et al. (2020)	China	Cell culture	-	-	HPN SK-N-SH HEK293T	BACE1-AS	Up	Cell Treatment, MTT Assay, LDH Cytotoxicity Assay, Cell Apoptosis Analysis, Western Blot Assay, qRT-PCR, Dual-Luciferase Reporter Assay	Accumulation of BACE1-AS reduces its protective activity by acting on miR-132-3p. Berberine interacts with BACE1-AS to up-regulate miR132-3p and protect neurons *via* the BACE1-AS/miR-132-3p axis.
Gu et al. (2020)	China	Cell culture	-	-	SH-SY5Y	RPPH1		MTT assay, qRT-PCR, Detection of apoptotic cells, Western blotting, Dual-luciferase reporter assay	Aβ25-35-induced apoptosis is attenuated by the RPPH1 effect along the miR-326/PKM2 axis.
Qasim et al. (2020)	China	Cell culture Animal study	-	APPswe/PS1ΔE9 double transgenic mice	SH-SY5Y	RPPH1	up	Cell culture and transfection, Cell viabilities, Flow cytometry, Caspase-3 activity, Measurement for Aβ, RT-qPCR, Western blot, Dual-luciferase reporter assay	Rpph1 lncRNA reduces apoptosis due to beta-amyloid by activating the Wnt/β-catenin pathway and targeting miR-122, while Rpph1 lncRNA and miR-122 are up-regulated in AD mice.
He et al. (2020)	China	Case-control Cell culture	AD patients (35 cases and 35 controls)	-	SK-N-SH SK-N-AS	BACE1-AS		Isoflurane Treatment, qRT-PCR, Cell Proliferation Assay, Flow Cytometry, Western Blot, Dual-Luciferase Reporter Assay	BACE1-AS acts as a sponge for miR-214-3p. BACE1-AS potentiates isoflurane-induced neurotoxicity by acting on miR-214-3p.
Hong et al. (2020)	China	Cell culture Animal study	-	SAMP8 and SAMR1 mice	HT22	ENSMUST0000015746 ENSMUST00000175096 ENSMUST00000083211 NR_040673 ENSMUST00000148940 ENSMUST00000137025		MWM Test, Microarray, RNA Labeling, Array Hybridization, qRT-PCR, GO and KEGG Analyses, AD Cell Models and Knockdown of lncRNAs by antisense oligonucleotide (ASO)	These dysregulated lncRNAs and their nearby genes can play an essential role in the pathogenesis of AD.
Huang et al. (2020)	China	Cell culture Animal study	-	APP/PS1 transgenic mice	HEK293T SH-SY5Y N2A-APPsw	NEAT1	up	RT-PCR Analysis, RNA Pull-Down Assay, RNA Immunoprecipitation, Western Blot, ATP Level and Cytochrome C Oxidase Activity	In ADs animal models, neat1 is up-regulated and, by interacting with NEDD4L, promotes ubiquitination of PINK1 and disrupts the PINK1-dependent autophagy process.
Kurt et al. (2020)	Turkey	Case-control	AD patients (23 cases and 33 controls)	-	-	TTC39C-AS1 lnc-AL445989.1-2 LINC01420 lnc-CSTB-1 LOC401557	Up (TTC39C-AS1 lnc-AL445989.1-2LINC01420) Down (lnc-CSTB-1LOC401557)	Microarray Hybridization, and Scan, Microarray Data Analysis, qRT-PCR Analysis	The first three lncRNAs showed increased expression, and the other two showed decreased expression in patients compared to controls. KEGG analysis showed a significant relationship between these lncRNAs and metabolic pathways.
Zhou B. et al. (2020)	China	Cell culture Animal study	-	APPswe/PSEN1dE9 doubly transgenic mice	HEK 293T	MALAT1 Meg3 Sox2ot Gm15477 Snhg1	Up (MALAT1 Meg3 Gm15477 Snhg1) Down (Sox2o)	IHC analysis, Western blot, qPCR, Morris water maze tests, Aβ42 oligomer preparation, MTT assay	MALAT1, Meg3, Gm15477, Snhg1 are up-regulated, and sox2ot is down-regulated in the absence of tet2, which regulates them and are the main lncRNAs in the formation of neurons.
Li et al. (2020)	China	Cell culture Animal study	-	BALB/c mice	Hippocampal neurons from neonatal BALB/c mice	TUG1		Morris water maze test, Hematoxylin-eosin staining, Nissl staining, TUNEL staining, Determination of SOD activity and MDA content, MTT assay, flow cytometry, RT-qPCR	In hippocampal neurons, knockdown of TUG1 limits apoptosis by raising miR-15a levels and inhibiting ROCK1 expression.
Liu et al. (2020)	China	Cell culture	-	hCMEC/D3 HBVP NHA	-	LINC00662		RT-qPCR, Western blot, Immunofluorescence assays, FISH, Chromatin immunoprecipitation assays, microarrays, RIP assays	LINC00662 increases the Blood-Brain Barrier's permeability by suppressing ELK4, and the TRA2A / LINC00662 / ELK4 network plays a vital role in BBB regulation.
Ma et al. (2020)	China	Animal study	-	APP/PS1 mice	-	LNC_000854 LNC_001450 LNC_000217 LNC_000233 LNC_001741 LNC_001678		RNA-seq, RT-qPCR	q-PCR-validated lncRNAs represent a group of lncRNAs in the network of ceRNAs involved in processes such as synaptic plasticity, regulation of amyloid-β (Aβ) -induced neuroinflammation, and memory.
Wang D. et al. (2020)	China	Case-control	AD patients (72 cases and 62 controls)	-	–	BACE1-AS	Up	Plasma collection and exosome isolation, Western blot analysis, TEM, RT-qPCR Acquisition of brain images.	Expression levels of lncRNA BACE1 AD patients were meaningfully increased compared with the controls, but there were no differences in the levels between patients with varying severity of dementia. Further to this, BACE1 AS levels combined with right entorhinal cortex MRI parameters may improve AD diagnosis accuracy.
Wang Q. et al. (2020)	China	Cell culture Animal study		BALB/c male mice	SH-SY5Y	WT1-AS		qRT-PCR, Western Blot, Flow cytometry, FISH, ChIP assay, RIP, AD modeling, Morris water maze test, TUNEL staining	Inhibition of WT1 expression by overexpression of WT1-AS can suppress the regulatory axis of miR-375/SIX4 and prevent neuronal apoptosis.
Zhang and Wang (2021)	China	Case-control Cell culture	AD patients (48 cases)	-	SH-SY5Y BV2 HEK293	MAGI2-AS3	Up	Dual-luciferase reporter assay, qRT-PCR MTT assay, ELISA	In Alzheimer's disease, the expression of MAGI2-AS3 increases, and miR-374b-5p expression decreases. Decreased MAGI2-AS3 expression and increased miR-374b-5p expression reduce Aβ-induced neurotoxicity and inflammation. The MAGI2-AS3/miR-374b-5p axis can be considered as a biomarker.
Xu et al. (2020)	China	Cell culture	-	-	SH-SY5Y SK-N-SH	SOX21-AS1	Up	qRT-PCR, Cell viability assay, Flow cytometry, Western blot, Dual-luciferase reporter assay, RIP assay	SOX21-AS1 expression increased in Aβ1-42-treated cells, and miR-107 expression decreased. Silencing of SOX21-AS1 by sponging miR-107 reduces nerve damage caused by Aβ1-42.
Yan et al. (2020)	China	Cell culture Animal study	-	APP/PS1 double transgenic mice	SH-SY5Y	linc00507	Up	qRT-PCR, Western blot, FISH, Luciferase reporter assay	Expression of linc00507 is elevated in the Alzheimer's disease model. The MAPT and TTBK1 genes are the direct targets of miR-181c-5p. By binding to miR-181c-5p as a ceRNA, linc00507 inhibits miR-181c-5p and increases the expression of its target genes involved in tau phosphorylation.
Zhao et al. (2020)	China	Cell culture Animal study	-	mice C57BL/6 APPswe/PS1dE9 double transgenic mice	SH-SY5Y	NEAT1	Up	qPCR, Cell culture, ChIP assay, RIP-qPCR	Increased NEAT1 expression can play a neuroprotective role and regulate microtubule stability by affecting the FZD3/GSK3β/p-tau axis.
Zhou B. et al. (2020)	China	Cell culture	-	-	Pc12	ANRIL		Cell culture, RT-qPCR, CCK-8 assay, Luciferase reporter assay	ANRIL reduces the expression of miR-125a by binding to it. In the Alzheimer's disease model, ANRIL silencing increases neurite outgrowth and suppresses cell apoptosis and inflammation.
Zhou Y. et al. (2020)	China	Case-control Cell culture Animal study	AD patients (18 cases and 18 controls)	APP/PS1 mice	SH-SY5Y	BACE1-AS	Up	qRT-PCR, western blot, Cell culture, HE staining and a TUNEL assay, IHC	BACE1-AS expression is increased in Alzheimer's disease. On the other hand, the autophagic activity also increases in the model of Alzheimer's disease. BACE1-AS indirectly reduces ATG5 expression by miR-214-3p. BACE1-AS silencing reduces neuronal damage and autophagy by affecting the miR-214-3p/ATG5 axis.
Zhuang et al. (2020)	China	Case-control	AD patients (120 cases and 120 controls)	-	-	MALAT1	Up	RT-qPCR	In AD patients, MALAT1 expression increased, and its target expression, miR-125b, decreased. MALAT1 and miR-125b may be involved in disease management through interaction with FOXQ1, PTGS2, and CDK5 genes.
Yue et al. (2020)	China	Cell culture animal study	-	AD mice (2vo)	N2a mouse neuroblastoma cells	XIST		qPCR, Immunofluorescence assay, Western blot assay, Aβ1–42 detection	The shutdown of lncRNA XIST attenuates the function of BACE1 in the progression of AD through miR - 124 and can be considered a target for treatment.
Ma et al. (2019)	China	Cell culture Animal study	-	C57BL/6J mice	SH-SY5Y 20E2	BACE1-AS		RNA interference, RT-qPCR, Extraction of cell and total proteins from brain tissues, Western blot analysis, ELISA	BACE1-AS is involved in regulating BACE1 expression and Aβ production in APPsw transgenic cells.
Bastard et al. (2020)	China	Cell culture, animal model	-	Sprague Dawley rats	PC12 C6	MALAT1	Up	Morris water maze training, Hematoxylin and eosin (HE) staining, Microarray analysis, Flow cytometry, ELISA, Western blot	The ability of MALAT1 was determined in neuronal recovery following the occurrence of AD *via* the miR-30b/CNR1 axis and the PI3K/AKT pathway.
Banerjee et al. (2021)	Israel	Animal study	-	Zebrafish	-	MSTRG.1987 MSTRG.28608 MSTRG.535 MSTRG.70 MSTRG.26654 MSTRG.17001 MSTRG.23990 MSTRG.12623 MSTRG.1031 MSTRG.8212 MSTRG.5861 MSTRG.24808		Library construction and sequencing, Transcriptome assembly, RT-qPCR	Hypoxia causes differential expression of genes associated with Alzheimer's disease (AD). Several new lncRNAs were similar in the synthetic regions of zebrafish and human brains, and eight functional lncRNAs related to the expression of Alzheimer's genes were examined.
Zhang et al. (2021)	China	Cell culture Animal study	-	AD mice	PC12	H19	Up	Luciferase reporter assay, RNA-pull down assay, RT-qPCR	Silencing of H19 is associated with elevated miR-129 levels, improves survival, and suppresses Aβ25-35-induced apoptosis in PC12 cells.

## Discussion

### Expression Regulation, the Most Crucial Step in the Process of LncRNA Function

The precise expression of LncRNAs is vital because their expression is low compared to the genes encoding proteins and they are much less expressed than them. The specificity of tissue expression and low expression means that the expression of LncRNAs must be highly regulated (Hansen et al., 2011). Remarkably, as much as the genes encoding proteins are sensitive to developmental conditions and environmental stress changes, these changes affect LncRNAs (Cawley et al., 2004; Yang et al., 2013). On the other hand, because LncRNAs themselves are involved in regulating the expression of other genes, small changes in their expression can manifest as a significant milestone in the expression regulating of other genes, disrupting the co-expression network between LncRNAs and mRNAs (Lim et al., 2019). There have not been many studies on the mechanisms of regulation of lncRNA expression. However, a few can be mentioned, including chromatin state, which can be extensively altered by DNA methylation and histone modification. Promoter hyper-methylation in the MEG3 lncRNA gene causes downregulation of expression, which is increased by interfering with DNA methyltransferase activity (Braconi et al., 2011). In addition, methylated cytosines are found in critical functional regions of LncRNAs such as XIST and HOTAIR and show their effect on the function of LncRNAs (Amort et al., 2013).

The effects of histone acetylation on the chromatin state, which prevents the formation of its super-condensing structure and facilitates the expression of surrounding (lncRNA)genes, can also be mentioned as LncRNAs expression regulation mechanisms (Chen and Pikaard, 1997). The high sensitivity of regulating the expression of LncRNAs, tissue specificity, and their essential and indispensable roles in regulating the expression of other genes predispose them in the case of dysregulation to the pathogenesis of various diseases, including neurodegenerative disorders, in particular, AD. Among these, GWAS studies identify the potential of several LncRNAs in the pathogenesis of AD by examining many polymorphisms. One study discovered eight variants in lncRNA genes that had never been studied before in AD. These polymorphisms can result in changes in lncRNA secondary structures, resulting in the loss or increase of microRNA binding sites (miRNAs) and downstream pathway regulation (Kretzschmar et al., 2021). According to the present study, the significant contribution of dysregulated LncRNAs in AD is assigned to Bace1-AS, NEAT1, MALAT1, SNHG1, 17A, and Rpph1 LncRNAs, respectively. Among the reported dysregulation of lncRNA expressions, the significant share of these dysregulations with 56% is assigned to the up-regulated, and 6.9% of the total cases reported are down-regulated, and 37.5% of the studies They also did not report up or down-regulation of LncRNAs. Interestingly, the scales in these dysregulations of expressions in AD weigh heavily toward up-regulation, and down-regulated ones are meaningfully less.

### LncRNA Authority in Transcription Regulation

**Figure 3:**
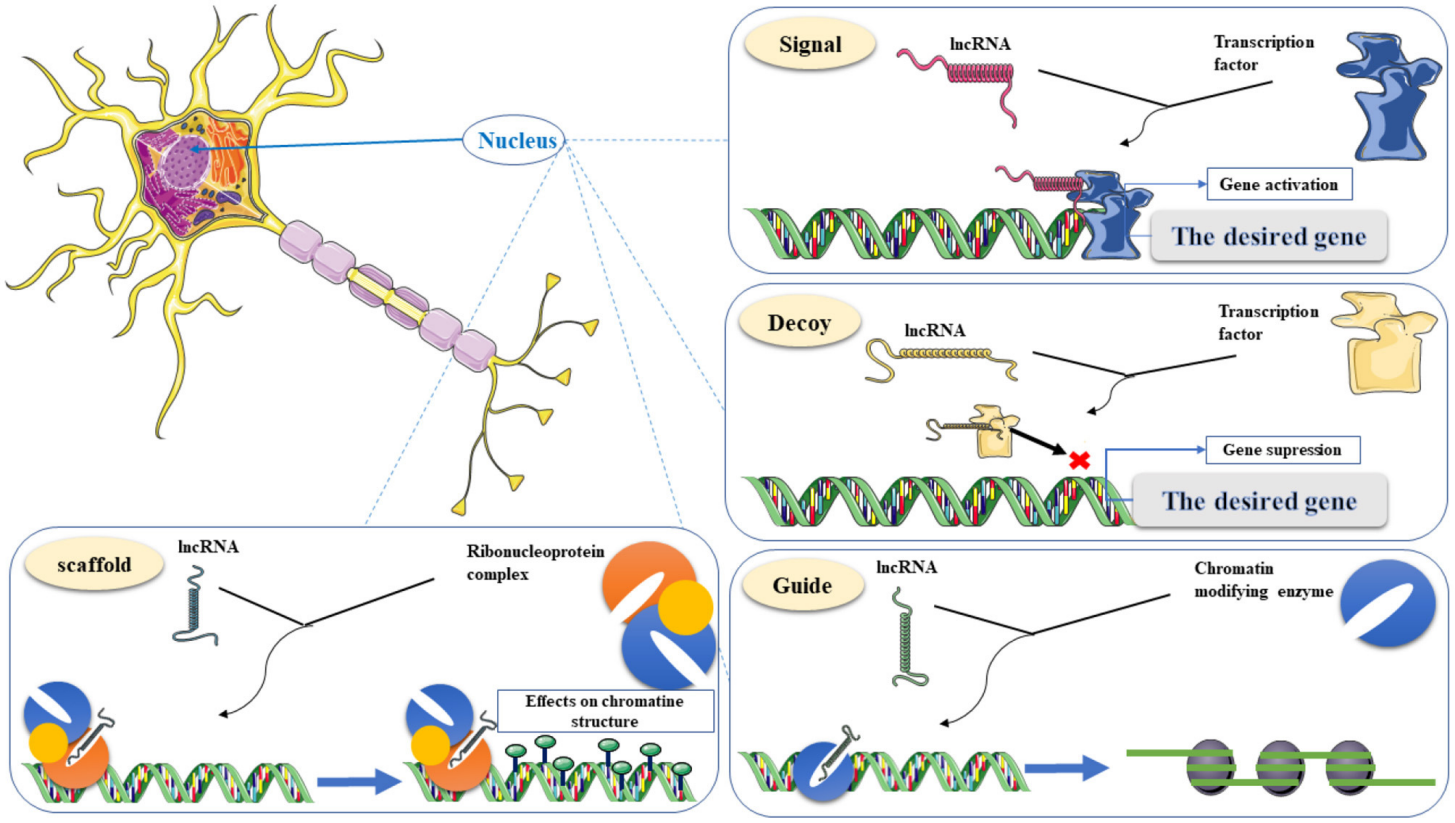
Classification of lncRNA functions in gene transcriptional regulations, including Signal, Decoy, Guide, and Scaffold. Signal, recruiting transcriptional proteins to the target gene to enhance gene expression. Decoy, a “molecular sink” for RNA-binding proteins (RBPs). Guide, binding to chromatin-modifying enzymes and direct to the target for epigenetic modification. Scaffold, a nest for connecting several effective partners and transporting them simultaneously to one place. This graphical figure was created using the vector image bank of Servier Medical Art (http://smart.servier.com).

One of the essential functional areas of LncRNAs is gene expression regulation. LncRNAs affect gene expression through various molecular mechanisms. Some lncRNAs can function simultaneously through several of these mechanisms, so these mechanisms cannot be considered in isolation. LncRNAs can act as guides, signals, decoys, and scaffolds (Wang and Chang, 2011). As a guide, lncRNAs can bind to proteins such as chromatin-modifying enzymes and direct them to their specific target and mediate epigenetic modification. In this mechanism, lncRNAs can change the pattern of gene expression in cis or trans. LncRNAs such as ANRIL, XIST, HOTAIR, and KCNQ1OT1 can serve as chromatin modifier enzymes to reprogram epigenetic status (Bhat et al., 2016). LncRNAs can also act as molecular signals to change chromatin structure and recruit transcriptional proteins to the target gene to enhance gene expression (Wang and Chang, 2011; Bhat et al., 2016). Functional flexibility in the structure of LncRNAs as a decoy mechanism provides the ability to act as “molecular sinks” for RNA-binding proteins (RBPs), including transcription factors, regulatory factors, and chromatin modifiers and these groups of lncRNAs are likely to be negative regulators. Also, in this mechanism, lncRNAs sponge miRNAs in a ceRNA network and prevent them from binding to the target RNA (Wang and Chang, 2011). miRNAs bind to the 3'UTR sequences or the coding sequences in mRNA molecules, reducing mRNA stability and the abundance of target proteins (Baek et al., 2008; Bartel, 2009). Scaffolds as a nest for connecting several effective partners and transporting them simultaneously to one place can be considered one of the capabilities of LncRNAs in the transcription process. These molecular companions can activate or suppress transcription (Wang and Chang, 2011; Bhat et al., 2016). The following is a schematic of the four regulatory mechanisms in the transcription regulation process (Figure 3). Because lncRNAs are involved in various human diseases which AD can be considered one of the main ones, knowing the mechanism of action and their characteristics facilitate their application in targeted diagnostics, monitoring progression, and treatment (Bhat et al., 2016). The following section provides a comprehensive overview of up and down-regulated LncRNAs.

## Conclusion

In addition to regulating the expression of other genes, LncRNAs play critical regulatory roles by interacting with miRNAs in the ceRNA network. Tissue expression specificity is another factor that makes LncRNAs more sensitive. Low expression of LncRNAs compared to other genes and their essential role in vital cell mechanisms causes the slightest change or dysregulation in the expression of LncRNAs as a disorder, especially neurodegenerative diseases. Among these, AD can be considered the most important member of this group of diseases that LncRNAs also play an important role in its etiology due to the tissue expression specificity of 40% of them related to the brain. So far, many studies have examined the expression of LncRNAs in AD. In this review, we tried to provide a comprehensive summary of studies that have used validated molecular methods and provide an overview of the role of LncRNAs in the pathogenesis of this disease. The same project could be carried out in other neurodegenerative diseases, such as Parkinson's or ALS, and the role of LncRNAs in it can be discussed. On the other hand, further studies on the existing pathways for each of the mentioned LncRNAs have sound potential. Finally, it is decent to mention that there were some limitations to our study. First, we can mention the searching process. During it, all efforts were focused on selecting the keywords to cover the studies on the subject entirely. On the other hand, during screening studies, a study may be lost. It should also be noted that there were several studies that, despite much effort, could not provide their full text (Yang et al., 2018).

## Data Availability Statement

The original contributions presented in the study are included in the article/supplementary material, further inquiries can be directed to the corresponding author/s.

## Author Contributions

MT, MR, and SG-F wrote the draft and revised it. MA, MH, SK, HS, MM, and MK designed the tables and figures and collected the data. All the authors read and approved submitted version.

## Funding

The research protocol was approved and supported by the Molecular Medicine Research Center, Tabriz University of Medical Sciences (grant number: 67060).

## Conflict of Interest

The authors declare that the research was conducted in the absence of any commercial or financial relationships that could be construed as a potential conflict of interest.

## Publisher's Note

All claims expressed in this article are solely those of the authors and do not necessarily represent those of their affiliated organizations, or those of the publisher, the editors and the reviewers. Any product that may be evaluated in this article, or claim that may be made by its manufacturer, is not guaranteed or endorsed by the publisher.
